# Biocompatible and Antibacterial Chemical Coatings on TiZr Dental Implants

**DOI:** 10.3390/jfb16030112

**Published:** 2025-03-20

**Authors:** Vlad Gabriel Vasilescu, Toma Lucian Ciocan, Andreea Mihaela Custura, Florin Miculescu, Miruna Stan, Ionela Cristina Voinea, Dumitru Dima, Florentina Ionela Bucur, Andreea Veronica Dediu-Botezatu, Marian Iulian Neacșu, Elisabeta Vasilescu, Marina Imre

**Affiliations:** 1 Discipline of Dental Prosthesis Technology, Faculty of Dentistry, “Carol Davila” University of Medicine and Pharmacy, Dionisie Lupu Street, No. 37, District 2, 020021 Bucharest, Romania; vlad.vasilescu@umfcd.ro; 2 Department of Metallic Materials Science, Physical Metallurgy, National University of Science and Technology, Politehnica Bucharest, 313 Splaiul Independentei, J Building, District 6, 060042 Bucharest, Romania; f_miculescu@yahoo.com; 3 Department of Biochemistry and Molecular Biology, Faculty of Biology, University of Bucharest, 91-95 Splaiul Independentei, 050095 Bucharest, Romania; miruna.stan@bio.unibuc.ro (M.S.); ionela-cristina.voinea@bio.unibuc.ro (I.C.V.); 4 Department of Chemistry, Physics and Environment, Faculty of Science and Environment, “Dunărea de Jos” University, Domnească Street, 111, 800201 Galați, Romania; dimadumitru@yahoo.com (D.D.); andreea.botezatu@ugal.ro (A.V.D.-B.); 5 Department of Food Science, Food Engineering and Applied Biotechnologies, Faculty of Food Science and Engineering, “Dunărea de Jos” University, Domnească Street, 111, 800201 Galați, Romania; florentina.bucur@ugal.ro; 6 Department of Materials and Environmental Engineering, Faculty of Engineering, “Dunărea de Jos” University, Domnească Street, 111, 800201 Galați, Romania; marian.neacsu@ugal.ro (M.I.N.); elisabeta.vasilescu@yahoo.com (E.V.); 7 Discipline of Prosthodontics, Faculty of Dentistry, “Carol Davila” University of Medicine and Pharmacy, 37 Dionisie Lupu Street, District 2, 020021 Bucharest, Romania; marina.imre@umfcd.ro

**Keywords:** dental implant, silver, antibacterial inhibition zone, chitosan, biocompatibility, human osteoblasts, human gingival fibroblasts

## Abstract

This research aims to study the antibacterial coatings of invasive surgical medical devices, including dental implants, to reduce superficial and deep local infections over the long term. To obtain the coating without altering the initial properties of the substrate (dental implant made of TiZr bioalloy), simple, cost-effective, and efficient methods were employed, such as chemical deposition of silver (Ag). The deposition characteristics were analyzed using scanning electron microscopy (SEM), EDX analysis, and FT-IR infrared analysis. The in vitro testing of antimicrobial activity was conducted using the diffusion method by cultivating the bacterial strains Escherichia coli (*E. coli*) ATCC25922 and Staphylococcus aureus (*S. aureus*) ATCC25923 and measuring the diameter of the bacterial inhibition zone. Investigations and biocompatibility evaluations were performed on both uncoated and silver-coated (Ag) samples by analyzing cell viability and morphology in the presence of human fetal osteoblasts (hFOB cell line) and human gingival fibroblasts (HFIB-G cells) after 8 days of incubation. The research results confirm the biocompatibility of the coating, demonstrated by the lack of significant differences in cell density between the Ag-coated samples and the control group, as well as by the fact that the silver-coated surface effectively supports actin cytoskeleton organization, adhesion, and migration of both human osteoblasts and gingival fibroblasts. The results regarding the antibacterial efficiency of the silver implant coating indicated that the *E. coli* bacterial strain is more resistant than *S. aureus*. The resistance difference between the two bacterial strains was attributed to differences in the structure of their cell envelopes.

## 1. Introduction

The resistance of pathogenic bacteria to antimicrobial agents manifested in recent years is a major health problem [1]. Medical devices such as endotracheal tubes, vascular and urinary catheters, and hip prostheses are responsible for more than half of nosocomial infections in the U.S. [2]. Depending on the degree of risk of infection, dental implants are considered critical medical devices which require the observance and maintenance of sterile conditions, starting with the manufacturing stage of the implant and continuing with the invasive surgical act of implantation [3,4]; ensuring an aseptic environment at implantation is a mandatory condition to avoid any infection, which could later be the cause of implantation failure or even the danger of serious diseases.

Since the materials used in the manufacture of dental implants are not effective in the process of microbial inhibition, there is the solution of coating the implant with metals which confer antimicrobial properties in order to prevent infections. Nanoparticles such as silver, copper, and zinc oxide possess antimicrobial properties, making them effective in combating microorganisms. Applying these nanoparticles to the surface of a dental implant can effectively discourage bacterial accumulation and reduce the risk of infectious complications [5]. Enhancing the antibacterial capability of the implant surface aims to prevent peri-implant diseases, thereby minimizing clinical failure [6]. Among metallic nanoparticles, silver nanoparticles are known to have a broad spectrum in antimicrobial protection against Gram-positive and Gram-negative bacteria, fungi, and certain viruses, including antibiotic-resistant strains [7,8]. Used since ancient times as an antimicrobial agent, silver is widely recommended in the medical field [4,8], the best known being silver-impregnated medical devices, surgical masks, and implantable devices that have demonstrated significant antimicrobial efficacy.

The antimicrobial mechanism of silver, which is not yet fully understood, has been better elucidated with the advancement of modern analytical methods, allowing direct observation of morphological and structural changes caused by silver ions on bacteria [9,10,11]. The inhibitory effect of silver nanoparticles arises from their adsorption onto the bacterial cell wall, inactivation of cellular enzymes, and disruption of membrane permeability. The effectiveness of silver in antibacterial protection lies in its highly reactive ionized form [12,13].

Silver ions interact both with the cell membrane surface, which consists of sulfur-containing proteins, and with the nucleus, where they associate with the bacterial cell’s DNA [11,14,15,16]. Once inside bacterial cells, silver ions induce changes such as the inactivation of respiratory enzymes in the cytoplasmic membrane, disruption of DNA replication and prevention of cell multiplication, generation of reactive oxygen species and free radicals, and inhibition of molecular signal transduction pathways [9,12,13,16,17,18,19,20,21,22,23,24,25]. These changes result in the inactivation of key enzymes necessary for cellular “respiration” [24] or even lead to cell death [25].

In antibacterial coatings, silver can be delivered in various forms, but the effective forms of silver that can achieve microbial inhibition are silver salts. Among these, silver nitrate (AgNO_3_) has proven to be the most effective due to its continuous release of a moderate amount of silver ions and its ability to control the physicochemical parameters that influence the antimicrobial potential of silver nanoparticles, primarily size, shape, stability, concentration, and colloidal state [14,26,27,28,29].

Small nanoparticles with a large specific surface area and a high number of atoms will exhibit a higher level of antimicrobial activity compared to bulk silver metal [30,31,32]. Due to better contact with microorganisms, smaller nanoparticles have a greater bactericidal effect than larger particles [32].

At the same time, they can also trigger a cytotoxic signal [33,34,35,36]. The toxicity of silver nanoparticles at a high concentration can most likely be attributed to their increased surface area and the release of protein-binding ions and nucleic acids interfering with their functions. Ag nanoparticles are toxic to both microorganisms and human cells. That is why much of the research aims to develop methods to reduce the toxicity of nanoparticles, which can be achieved by combining silver with other polymeric and ceramic substances [37,38,39,40]. Yu et al. developed hybrid lysozyme/chitosan/silver/HA coating materials on a titanium surface and demonstrated that the toxic properties of silver were reduced by chitosan, without affecting the antibacterial properties and stability of silver nanoparticles [40].

Current studies highlight the necessity of designing implant surfaces to ensure long-term osseointegration without infections, considering that the complications caused by infection reach up to 40%, a problem regarded as one of the most common and difficult to treat issues [6].

Zhao et al. reviewed in vitro and in vivo investigations of antibacterial coatings on titanium-based implants. They concluded that although progress has been made, information on in vivo testing and clinical use of implants remains limited [41]. Additional issues related to the methodological quality and experimental models used in some studies, the small number of results reported in the literature, variability in experimental models, and lack of consistency between studies are further arguments supporting the conclusion that the study of antibacterial efficiency on titanium substrate surfaces remains a significant challenge [42,43,44,45,46].

In this context, research analyzing the antibacterial efficiency of chemically deposited silver on titanium-based alloy implants (TiZr), as covered in our study, can provide a foundation for discussion, both from the perspective of the methodology used and the results obtained. The study results can contribute to expanding knowledge regarding in vitro biocompatibility testing of coatings and the evaluation of antibacterial activity to determine the antibacterial protection zone.

The study reconfirms the necessity of continuing research to establish a conclusive database aimed at clarifying aspects such as determining the toxicity threshold of silver, the efficacy of adding chitosan in reducing toxicity, and evaluating the durability of antibacterial coatings for implantable medical devices.

## 2. Materials and Methods

**Figure 1:**
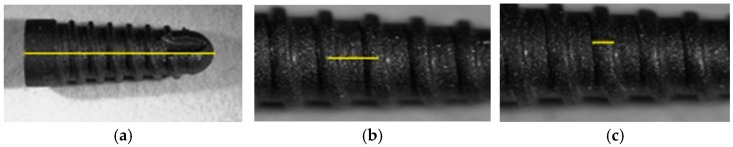
Dental implant (Ti10Zr) of self-tapping screw type (dimensional characteristics: (**a**) 8 mm, (**b**) 0.8 mm, (**c**) 0.2 mm).

The materials used in the experiments are self-drilling, self-tapping screw-type dental implants (Figure 1) made of Ti10Zr alloy (Patent RO No. 132079/2019), with an alumina-blasted surface (Tehnomed SRL, Bucharest, Romania).

The chemical coating was achieved by immersing the implants in prepared solutions. Tollens’ reagent and saturated solution of chitosan were used (2% acetic acid solution in which 10% chitosan was dissolved).

In the experimental research, we proceeded to heat the solution to temperatures of about 50 °C, maintained for about 10 min with stirring and additions of formic aldehyde in drop. Small silver particle deposits with a high degree of compactness and dispersion were obtained. The characterization of the sample’s surface (Ti10Zr implants) with and without chemical deposition was performed by scanning electron microscopy analysis (SEM, EDS and FT-IR infrared). The infrared spectra were collected using a Nicolet IS50 FT-IR spectrometer (Thermo Scientific, Waltham, MA, USA) equipped with a built-in ATR accessory, DTGS detector, and KBr beam splitter. Implants were analyzed using this method, similar to other studies in the literature [47,48,49,50,51], to identify the functional groups from the coatings. A total of 32 scans were added in the range 4000–400 cm^−1^ with a resolution of 4 cm^−1^. The air was taken as a reference for the background spectrum before each sample. After each spectrum, the crystal was cleaned with ethanol solution. To verify that no residue remained from the previous sample, a background spectrum was collected each time and compared with the previous background spectrum. The FT-IR spectrometer was placed in a room with a controlled atmosphere and constant temperature (21 °C).

The bacterial strains Escherichia coli ATCC 25922 and Staphylococcus aureus ATCC 25923 were used to test the antimicrobial activity of the solutions. The bacteria were maintained at −20 °C in culture medium supplemented with 30% glycerol. Cell activation was achieved by streaking on agar culture medium (1.5% *w*/*v*) and incubation at 37 °C for 48 h. *S. aureus* and *E. coli* were later cultured in BHI Infusion (Oxoid, Basingstoke, Hampshire, UK) and Luria Bertani, LB (Sigma-Aldrich, Steinheim, Germany), respectively. Cultures were obtained by transfering a bacterial colony to 10 mL of suitable culture medium using a sterile loop, followed by static incubation at 37 °C for 18 h. To determine the number of colony-forming units (CFUs), decimal dilutions of bacterial cultures were performed in saline phosphate buffer (PBS; pH 7.4) and 10 μL of each and inoculation by spotting on solid culture medium. The plates were incubated at 37 °C until the next day and the colonies were counted to determine the concentration of bacterial cells in the cultures. The bacterial strains, cultured according to the method described above, were inoculated in 7 mL of semi-solid Mueller Hinton (MHA, 0.75% *w*/*v* agar; Scharlau, Barcelona, Spain) tempered at 42 °C, at a concentration of ~10^7^ CFU (colony-forming units). The inoculated culture medium was homogenized and spread evenly in Petri dishes containing solid MHA (1.5% *w*/*v* agar). The plates were kept in a biosafety cabinet, in a sterile air stream, until the solidification of the inoculated culture medium. Subsequently, with the help of a sterile glass tube, wells with a diameter of 8 mm were cut. The samples to be analyzed were loaded into wells at a volume of 100 μL. The plates prepared in triplicate were incubated at 37 °C until the next day and the antimicrobial activity of the samples was assessed by measuring the total diameter of the inhibition zones in mm with a digital caliper (Burg Wächter, Wetter, Germany), from which the diameter of the wells was subtracted. The statistical analysis of the experimental data on antimicrobial activity was performed using the GraphPad Prism program, version 8 (GraphPad Software Inc., San Diego, CA, USA), by applying the Student test “*t*”. The differences were considered statistically significant for *p* < 0.05.

For the measurement of in vitro biocompatibility, human osteoblasts and human gingival fibroblasts were used. Human osteoblasts (hFOB 1.19 cell line—CRL-11372, purchased from ATCC, Manassas, VA, USA) were maintained at a temperature of 34 °C, in a humidified atmosphere, with 5% CO_2_, in Dulbecco’s modified Eagle’s medium (DMEM)/Ham’s F-12 medium without phenol red (1:1; Sigma, Darmstadt, Germany), supplemented with 10% fetal bovine serum (FBS), 2.5 mM of L-glutamine (Sigma), and 0.3 mg/mL of antibiotic G418 (Sigma). Human gingival fibroblasts (cell line HFIB-G, catalog number 1210412, Provitro AG, Berlin, Germany) were grown in DMEM with 10% FBS, at 37 °C, in an atmosphere humidified with 5% CO_2_. The cells were added at a density of 2 × 10^4^ cells/cm^2^ on the surface of the test samples, previously sterilized for 2 h under UV light, and on the plastic surface of a tissue culture plate with 6 wells, which served as the control of the experiments. The biocompatibility tests were performed after 8 days of incubation under standard conditions, according to the complete description by Vasilescu V.G. [52]. Actin filaments were observed by staining with 20 μg/mL FITC-phalloidin for one hour, after the cells were fixed and permeabilized. The nuclei were stained with DAPI for 15 min in the dark, and the visualization was made under an Olympus IX71 inverted fluorescence microscope (Olympus, Tokyo, Japan).

## 3. Results

### 3.1. Results Obtained from Surface Analysis of Samples (SEM, EDX, FT-IR)

**Figure 2:**
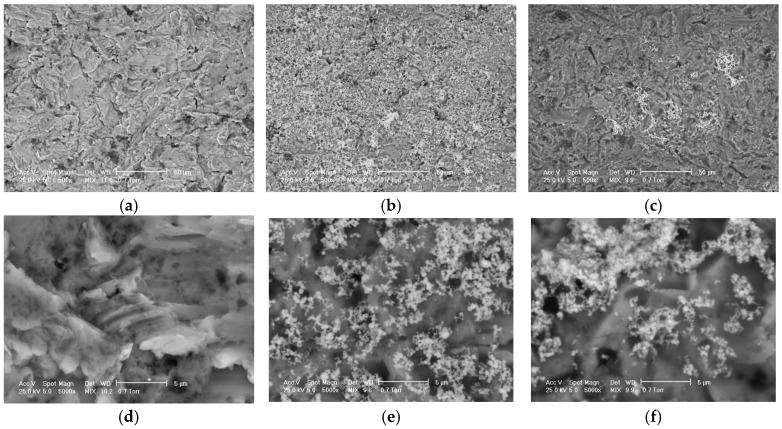
Sample surface morphology in initial state (**a**,**d**), with Ag deposition (**b**,**e**), and with Ag and chitosan (**c**,**f**). The micron bars are 50 µm (**a**–**c**) and 5 µm (**d**–**f**).

**Figure 3:**
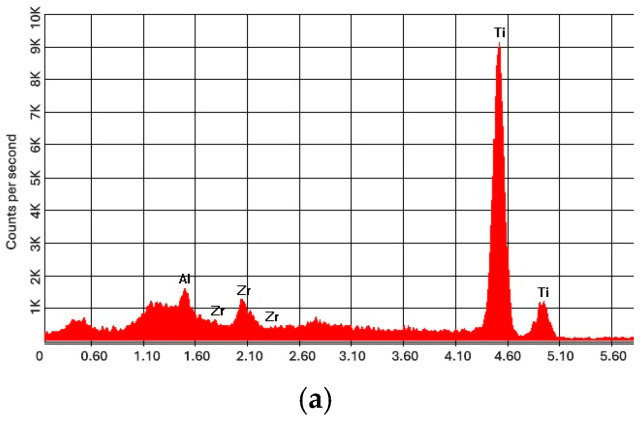

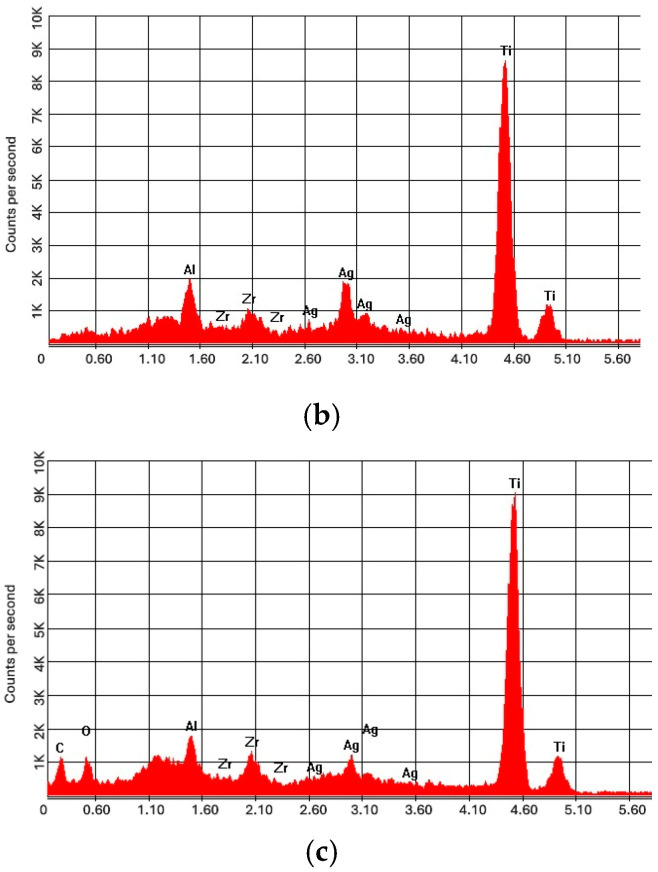
X-ray energy dispersive spectra of initial state (**a**), Ag-treated (**b**), and Ag and chitosan-treated samples (**c**). (Vertical axes represent intensity of the EDS signal in arbitrary units of CPS—counts per second; horizontal axes describe energy in keV).

**Figure 4:**
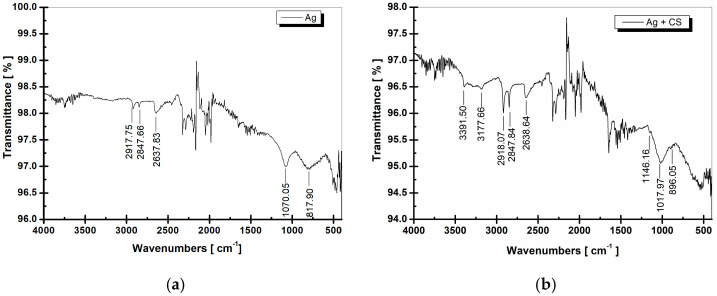
FT-IR analysis of the surface of implants with chemical deposition of (**a**) silver, (**b**) silver and chitosan.

**Figure 5:**
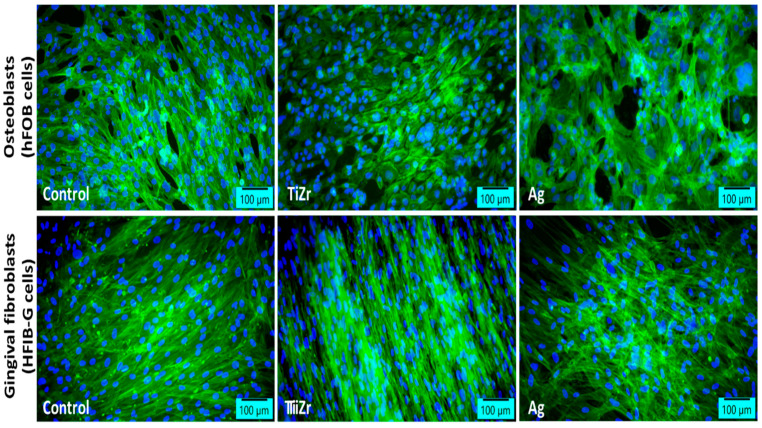
Fluorescent staining of actin filaments in human osteoblasts and human gingival fibroblasts after 8 days of incubation with Ag-coated samples (green: F-actin labeled with phalloidin-fluorescein isothiocyanate; blue: nuclei labeled with 4′,6-diamidino-2-phenylindole; scale bar: 100 μm).

The surface morphology of the samples in the untreated state (without coating), with silver coating, and with silver + chitosan coating is shown in Figure 2, high-lighting deposits with silver particles with small dimensions, with a high degree of compactness and dispersion. Energy dispersive spectroscopy analysis, EDX (Figure 3 and Table 1), quantifies the silver deposited on the implant surface. Figure 4 and Figure 5 show the infrared spectrum specific to the coating of the implant surface with silver and silver + chitosan.

For the sample coated with chitosan and Ag (Figure 4b), the peaks located at 2847 and 1647 cm^−1^ were attributed to aliphatic C–H symmetric stretching and C=O bonds, respectively. In the dual coating (Ag and chitosan), the appearance of (–OH) groups was observed at wavenumbers ranging from 3500 to 3200 cm^−1^ (OH stretch overlapped with NH stretch) [53,54]. The bands at approximately 3.170 and 1.640 cm^−1^ were assigned to amide I, while those at 1.566 and 1.312 cm^−1^ were assigned to amides II (-NH2 tensions) and III, respectively, from the chitosan chemical structure [53,54]. The band in the range of 3290 cm^−1^ was also observed, corresponding to the NH stretching region of chitosan. The band at 1017 cm^−1^ corresponded to C–O stretching [55]. The FT-IR results showed the presence of chitosan chemical deposition on the implant. In spectra from Figure 4b, vibration patterns indicating the presence of key functional groups including hydroxyl, carbonyl, and amine were observed.

The FT-IR analysis was performed on the flat area, positioned at the apical part of the implant (this portion has a flat shape as the implant structure is designed that way), directly on the ATR crystal. We acknowledge that the screw shape limits the possibility of analysis in other specific regions. However, this technique was employed to identify the characteristic bonds of the compounds used for the chemical deposition.

The presence of specific bands corresponding to the organic compound (chitosan) deposition was observed at the characteristic wavenumbers mentioned in the result interpretation (2847, 1647, and 3500 to 3200 cm^−1^). These bands are clearly distinguishable and do not appear in the spectra of Ag-coated or untreated implants. This preliminary FT-IR analysis, which confirms the chemical deposition and ensures reproducible results, complements the findings of subsequent analyses presented in the manuscript (e.g., SEM and EDS).

### 3.2. Results Obtained from in Vitro Analysis of the Viability and Morphology of Human Cells

Samples (Ti10Zr implants) uncoated and coated with Ag demonstrated good biocompatibility in the presence of human fetal osteoblasts (hFOB cell line) and human gingival fibroblasts (HFIB-G cells) after 8 days of incubation, evidenced by actin filament staining (Figure 5).

### 3.3. Antimicrobial Activity Testing Results

**Figure 6:**
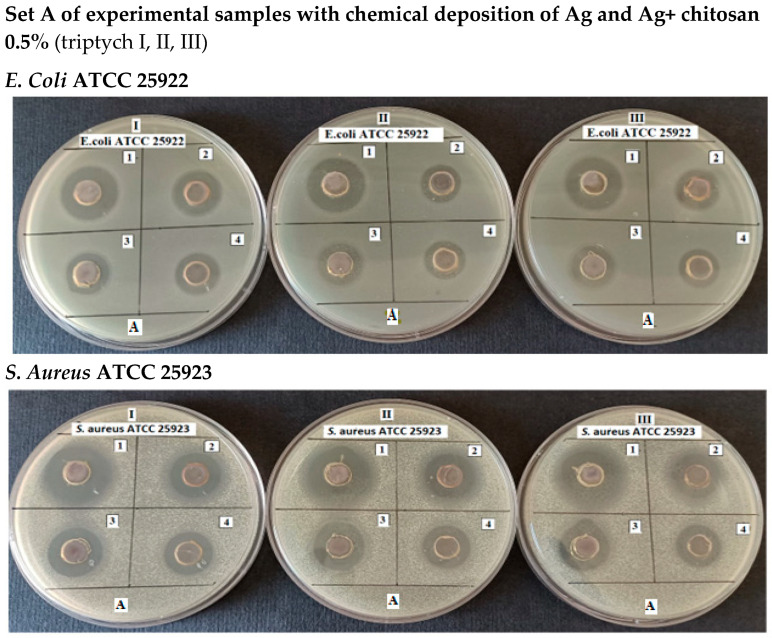
Formation of inhibition zones by the samples to be analyzed on MHA inoculated with *E. coli* ATCC 25922 and, respectively, *S. aureus* ATCC 25923 (0.5% chitosan).

**Figure 7:**
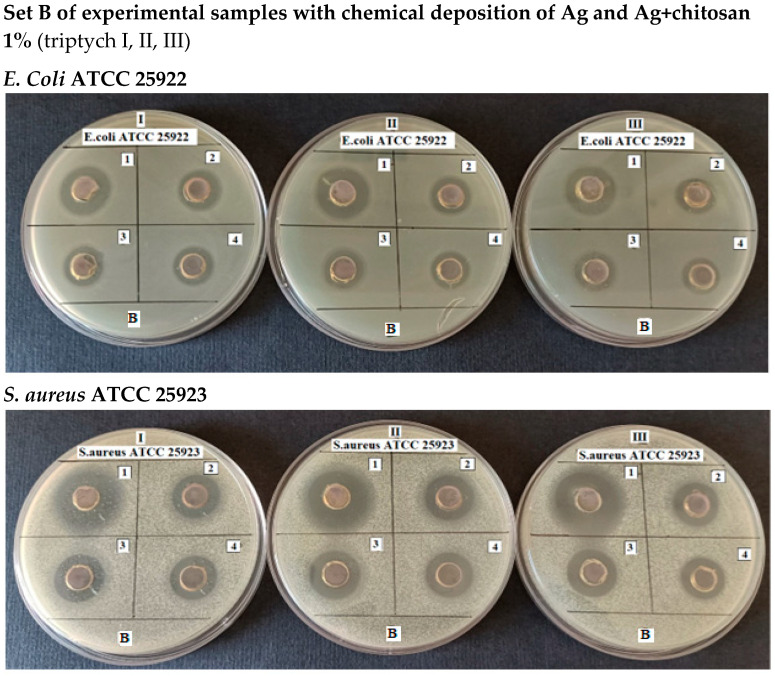
Formation of inhibition zones by the samples to be analyzed on MHA inoculated with *E. coli* ATCC 25922 and, respectively, *S. aureus* ATCC 25923 (1% chitosan).

The antimicrobial activity of the solutions was tested by applying the diffusimetric method, using *E. coli* and *S. aureus* as Gram-negative and Gram-positive model bacteria, respectively. The samples to be analyzed were loaded into wells at a volume of 100 μL and plates, prepared in triplicate, were incubated at 37 °C until the next day. The clear areas around the wells indicated inhibition of the growth of bacteria. The antimicrobial activity of the samples was assessed by measuring the total diameter of the inhibition zones from which the diameter of the wells was subtracted (Figure 6 and Figure 7, Table 2 and Table 3).

**Figure 8:**
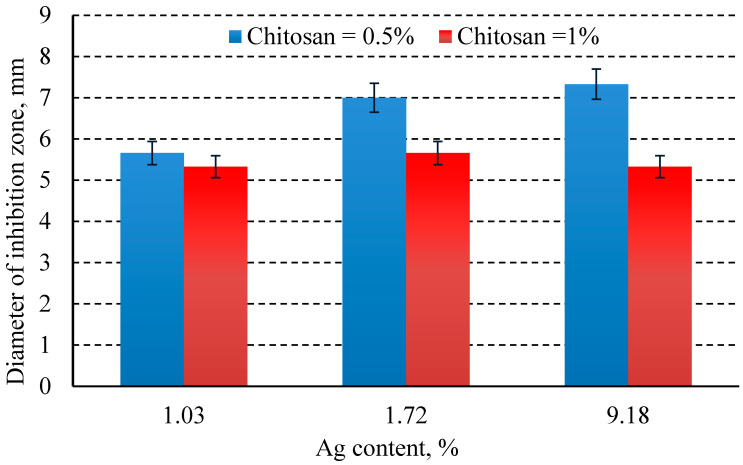
Diameter of the antimicrobial inhibition zone according to % Ag and % chitosan in *E. coli*.

**Figure 9:**
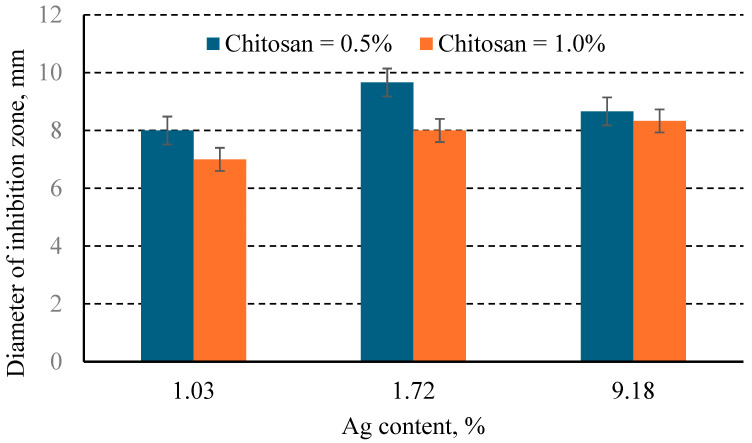
Diameter of the antimicrobial inhibition zone according to % Ag and % chitosan in *S. aureus*.

The influence of the concentration of silver in the chemical coating on the diameter of the bacterial inhibition zone, for different concentrations of chitosan, is shown in the figures below (Figure 8 and Figure 9).

Experimental data demonstrate that the antibacterial effect of silver, evaluated by the diameter of the inhibition zones formed on the culture medium inoculated with either *E. coli* or *S. aureus* around wells containing the tested solutions, increases with higher concentrations of silver, irrespective of the bacterial strain. At the same concentration, the antibacterial effect of silver varies depending on the bacterial strain, showing greater efficiency against *S. aureus* (*p* = 0.0006).

Measurements indicated that when the implant was coated solely with silver, the maximum inhibition zone diameter was 12 mm for *E. coli* and 15.33 ± 0.57 mm for *S. aureus*. As anticipated, in the case of silver + chitosan coatings, the diameter of the inhibition zones decreased as the silver concentration decreased, for both bacterial strains. It was also observed that *S. aureus* was more sensitive to the antimicrobial action compared to *E. coli* (*p* < 0.05).

For the same silver concentration in the coating but varying chitosan concentrations, the study revealed the following:

- At low silver concentrations (approximately 1% Ag), increasing the chitosan concentration from 0.5% to 1% did not significantly affect the inhibition zone diameter for *E. coli* (*p* = 0.51) or *S. aureus*.
- At higher silver concentrations (above 1.7%, with the tested range being 1.72–9.18% Ag), increasing the chitosan concentration from 0.5% to 1% significantly influenced the inhibition zone diameter for *E. coli* (*p* = 0.01), but not for *S. aureus* (*p* = 0.11).

For coatings containing the same silver concentration (e.g., 9.18% Ag) but different chitosan concentrations (0.5% and 1%), the inhibition zone diameter for *E. coli* did not show significant differences (*p* = 0.05). Similarly, this observation was valid for *S. aureus* (*p* = 0.51).

## 4. Discussion

The results of this experimental study provide information regarding the effectiveness of antibacterial silver coatings, aimed at reducing local superficial and deep infections of dental implants made from titanium-based alloys. The data are based on tests measuring the diameter of the bacterial inhibition zone, by culturing *Escherichia coli* (*E. coli*) ATCC25922 and Staphylococcus aureus (*S. aureus*) ATCC25923 strains, and investigating the biocompatibility of the coatings using cell viability and morphology analysis, in the presence of human fetal osteoblasts (hFOB cell line) and human gingival fibroblasts (HFIB-G cells). The experimental study provides new insights regarding the treatment of titanium alloy surfaces used in implantology to improve cellular interaction. It presents a method for evaluating the biocompatibility of coatings, with results confirming its biocompatibility by showing that the actin cytoskeleton in both types of cells exhibited a well-organized structure, with numerous lamellipodia and filopodia forming cell-to-cell junctions, similar to those in the control. These observations confirm previous findings [56] which suggest that titanium-based surfaces coated with silver promote optimal cytoskeletal organization and effective cellular interactions, essential for osteointegration. No significant differences were observed in cellular density between silver-coated samples and the control. These findings are supported by other studies that highlight the fact that treating TiZr alloy surfaces significantly enhances cellular interaction and contributes to the clinical success of dental and orthopedic implants [57]. Silver coatings are known for their antibacterial properties, with low cytotoxicity to human osteoblasts in both in vitro and in vivo conditions [58]. Recent studies show that titanium-based alloys with silver coatings do not alter the viability of gingival fibroblasts after 3 days, compared to the control, although initially, during the first 24 h of incubation, they caused a decrease in cellular metabolic activity [59]. Significant results also concern the determination of the antibacterial inhibition zone size, revealing an interesting aspect related to the difference in resistance between the two bacterial strains, attributed to differences in their cell wall structures. The cell wall of Gram-positive bacteria consists of many layers of peptidoglycan (30–100 nm thick), while Gram-negative bacteria have a more complex cell wall, consisting of a thinner peptidoglycan layer (10 nm thick) surrounded by an outer membrane of lipopolysaccharide [60]. In this study, *E. coli* was found to be more resistant to the tested coatings than *S. aureus*. These results are consistent with other studies [38] which observed a higher susceptibility of *S. aureus* compared to *E. coli* to the antimicrobial action of silver–chitosan composite spheres, and those by some others [61], who discovered a similar behavior of bacteria in the presence of silver nanoparticles. On the other hand, there are reports indicating that *S. aureus* tends to be more resistant to AgNPs compared to *E. coli* [62,63]. Although the full mechanism of action of AgNPs against bacteria has not been fully understood, studies have attempted to provide some perspective. For instance, another study [64] proposed that AgNPs create “pits” in the outer membrane of Gram-negative bacteria and suppress the activity of membrane enzymes. In the case of Gram-positive bacteria, it was shown that AgNPs strongly bind to peptidoglycans, generating “pits” in the cell wall. As AgNPs bind to the underlying layers, more Ag+ is released, and the pits grow larger [65]. Interestingly, the study concluded that this phenomenon affects Gram-positive bacteria more than Gram-negative ones, as the Gram-positive cell wall has more peptidoglycan layers, which could explain the results of our study. The study also revealed the influence of the silver concentration in the coating, as well as the effect of chitosan present in the coating along with silver, on the diameter of the inhibition zones when testing on *E. coli* and *S. aureus* strains. The obtained results are considered preliminary (guiding) and will be used in future research as a starting point for further exploration of these aspects, including determining the optimal silver concentration in coatings with antibacterial efficiency for dental implants, establishing the toxicity limit of silver, and evaluating the durability of the coating, among others.

*E. coli* is a Gram-negative enteric bacterium commonly found in the intestines of humans and animals. In general, this bacterium is non-pathogenic in its normal habitat, but it can become pathogenic when it infects tissues outside the intestines [66,67]. Although rare, enteric bacteria, including *E. coli*, have been implicated in maxillary and mandibular infections following dental extractions, especially when combined with poor oral hygiene and systemic conditions such as diabetes, which can delay wound healing. Maxillary or mandibular osteomyelitis is generally caused by Staphylococcus aureus and Staphylococcus epidermidis; however, there are reports of *E. coli* or other enteric bacteria being identified as etiological agents of these severe infections [68,69,70]. These findings support the consideration of *E. coli* in the differential diagnosis of maxillary or mandibular osteomyelitis, particularly in patients with risk factors such as diabetes and poor oral hygiene.

The limitations of using *E. coli* as a model in this study, and consequently the limitations in interpreting the results, are as follows:

Strain-Specific Variability: This experimental system used only single strains from each bacterial species tested. However, it is well known that there can be significant genetic variability even among strains of the same species. The antimicrobial response and virulence characteristics of different *E. coli* strains may vary considerably, which means that the results obtained with this particular strain may not be fully generalizable to all *E. coli* strains involved in infections.

Limited Research on *E. coli* Virulence in Implant-Associated Infections: The virulence potential of *E. coli* in implant-related infections has not been widely studied, with reports being limited to a few isolated cases [66]. Although characterization of *E. coli* strains is necessary, only a single case of *E. coli* isolated from a deep surgical wound infection was found.

ExPEC Strains and Their Relevance to Implant Infections: The *E. coli* strain used in this study was selected based on its classification as an extraintestinal pathogenic *E. coli* (ExPEC). ExPEC strains are known to cause infections outside the gut, such as urinary tract infections, sepsis, and meningitis. However, their specific role in implant-related infections remains less explored. It was assumed that their virulence potential would be similar to other ExPEC strains, but further studies are required to confirm the clinical relevance of *E. coli* in such infections.

Despite these limitations, *E. coli* remains a widely accepted Gram-negative model in antimicrobial studies due to its well-characterized biology and established role in standardized antimicrobial testing. The combination of *E. coli* and Staphylococcus aureus in this study provides a useful comparative model for assessing the antibacterial activity of silver-based coatings against both Gram-negative and Gram-positive bacteria. Moreover, the in vitro testing model is complemented by biocompatibility evaluations, through the analysis of cell viability and morphology in the presence of human fetal osteoblasts, to assess the safety of silver for human cells in antibacterial coatings.

However, future research should expand upon these findings by including a broader range of *E. coli* strains and conducting in vivo studies to further characterize the virulence potential of *E. coli* in implant-associated infections.

To further refine this model, future studies could incorporate additional *E. coli* strains with varying antibiotic resistance profiles and virulence factors to assess a broader range of antimicrobial responses. Additionally, biofilm formation assays could be performed to better mimic the in vivo conditions of implant-associated infections. These enhancements would provide a more comprehensive understanding of the antibacterial effectiveness of the silver-based coatings.

The antimicrobial potential of silver particles deposited on a metal support is determined by a range of physicochemical parameters, among which size, shape, and concentration of particles have a significant influence. Some authors [71] reported that smaller particles, with a larger available surface area for interaction, have a stronger bactericidal effect [28,30,31]. It was reported that nanoparticles ranging from 1 to 10 nm in size attach to the bacterial cell membrane surface and drastically disrupt its proper function [72], while 25 nm nanoparticles have the highest antibacterial activity and are toxic to bacterial cells at concentrations below 1.69 μg/mL Ag [71]. The influence of particle shape was confirmed by studying bacterial growth inhibition by nanoparticles of different shapes [72]. Triangular-shaped nanoparticles inhibit bacterial growth at a silver content of 1 μg, spherical nanoparticles at a total silver content of 12.5 μg, while rod-shaped nanoparticles require a total silver content of 50–100 μg [73]. Recent studies [74] showed that cubic silver nanoparticles determined a lower minimum inhibitory concentration (37.5 µg/mL) when tested on *E. coli*, compared to spherical (75 µg/mL) and fibrous (100 µg/mL) shapes. These data complement similar observations made by some other authors [75,76], who concluded that the facets of nanocubes present a higher surface energy compared to other shapes, leading to increased reactivity. These parameters (size and shape) are controlled by the method used to synthesize silver nanoparticles. Various physical and chemical methods for synthesizing silver nanoparticles are described in the literature [77,78,79,80,81,82,83,84]. Among these, chemical methods, including chemical reduction using a variety of organic and inorganic reducing agents, are the most commonly used. These agents reduce silver ions (Ag+) and lead to the formation of metallic silver (Ag0) clusters in the form of colloidal silver particles [85,86]. Tollens’ synthesis method led to the formation of silver nanoparticles with controlled size in a one-step process [87,88,89]. The most effective forms of silver for microbial inhibition are silver salts, with silver nitrate (AgNO_3_) being the most common as it allows for the continuous release of a moderate amount of silver ions, with control over particle size and shape. Therefore, the main process parameters for the chemical deposition of metallic silver onto implant surfaces are considered to be the nature of the prepared solutions, temperature, agitation rate of the solutions, and duration. In our study, chemical deposition with silver was carried out as follows: 100 mL of 2% AgNO_3_ solution was treated with 50 mL of 5% NaOH solution, and the resulting precipitate was dissolved by adding 50 mL of 2% NH_3_ solution. The implants were introduced into a 250 mL vessel with Tollens’ reagent, the solution was homogenized using a magnetic stirrer at 500 rpm, and 30% formic aldehyde was added. The implants were then washed with distilled water and placed in a drying oven at 105 °C. Different chemical deposition regimes were experimented with, both with and without heating the solution, and with the addition of formic aldehyde in droplets. Chemical reactions involved in the Ag deposition are described in other works by the authors [90,91,92]. Surface morphology analysis of the implant confirmed the presence of silver particles in both unheated and heated solution deposition regimes at 500 °C and 700 °C, with formic aldehyde additions in droplets. It was observed that, at short durations (up to 5 min), the silver particles were very large (agglomerations) with a low degree of dispersion. Furthermore, it was noted that heating the solution to 700 °C did not change the size, shape, or dispersion of the metallic silver particles deposited on the implant surface compared to heating at 500 °C. In the experiments, we heated the solution to approximately 500 °C, maintaining it for around 10 min with stirring and the addition of formic aldehyde in droplets. The silver particles obtained were small in size, with a high degree of compactness and dispersion.

## 5. Conclusions

The most important observations of this experimental study confirm many of the results found in the literature, while also highlighting the need for further research to establish a conclusive database on determining the optimal concentration of silver in antibacterial coatings for implants, the toxicity threshold of silver, and the efficacy of adding chitosan to reduce toxicity, as well as assessing coating durability, among other factors.

The study presents a method for evaluating the biocompatibility of the coating by testing the viability and morphology of both osteoblasts and human gingival fibroblasts. The results obtained show no significant differences in cell density between the Ag-coated and control samples, confirming the biocompatibility of the silver layer. These data align with findings in the literature indicating that the treatment of titanium alloy surfaces used in implantology significantly improves cellular interactions, promoting proper osseointegration and ensuring the clinical success of implants.

Regarding the antibacterial effect of the silver deposited on the dental implant, the results confirm that this effect increases with higher concentrations of silver in the coating, regardless of the bacterial strain, among the two tested (*E. coli* and *S. aureus*).

At the same concentration, the antibacterial effect of silver differs with respect to the two bacterial strains tested, showing higher efficiency in the case of the *S. aureus* strain. For the silver + chitosan coating, the diameter of the inhibition zones decreases with the concentration of Ag in the solutions, both for *E. coli* and *S. aureus* testing.

Taken together, the results of this experimental study provide valuable practical information on the effectiveness of silver antibacterial coatings, aimed at reducing superficial and deep local infections in long-term invasive surgical medical devices. Compared to the most recent literature, the findings contribute to expanding knowledge on in vitro testing of coating biocompatibility, evaluating antibacterial activity, and understanding the influence of silver concentration and the presence of chitosan on the diameter of the bacterial inhibition zone when tested on *E. coli* and *S. aureus* strains.

## Figures and Tables

**Table 1:** Semiquantitative results obtained by EDS for initial state (a), Ag-treated (b), and Ag and chitosan-treated samples (c).

Elements	Samples					
	a.		b.		c.	
	Wt, %	At, %	Wt, %	At, %	Wt, %	At, %
C-K	-	-	-	-	0.3	1.03
O-K	-	-	-	-	9.25	23.77
Al-K	7.7	13.54	7.23	13.82	7.82	11.91
Zr-L	10.48	5.45	5.43	3.07	7.92	3.57
Ag-L	-	-	18.34	8.77	9.18	3.5
Ti-K	Rest	Rest	Rest	Rest	Rest	Rest
Total	100	100	100	100	100	100

**Table 2:** Diameter of the inhibition zones produced by the samples to be analyzed on the inoculated MHA plates.

Bacterial Strain	Sample No.	Sample Type	Diameter * of the Inhibition Zone, mm
*E. coli* ATCC 25922	1	Ag ^a^	12
	Ag + Chitosan 0.5%		
	2	Ag ^b^ + Chito	7.33 ± 1.15
	3	Ag ^c^+ Chito	7.0
	4	Ag ^d^ + Chito	5.66 ± 0.57
*S. aureus* ATCC 25923	1	Ag ^a^	15.33 ± 0.57
	2	Ag ^b^ + Chito	8.66 ± 0.57
	3	Ag ^c^ + Chito	9.66 ± 0.57
	4	Ag ^d^ + Chito	8.0

Chemical deposition of silver with the concentration in coating (^a^)—18.34%; (^b^)—9.1%; (^c^) −1.72%; (^d^) −1.03% and chitosan 0.5%. (*) Average ± StDev.

**Table 3:** Diameter of the inhibition zones produced by the samples to be analyzed on the inoculated MHA plates.

Bacterial Foreign	Sample No.	Sample Type	Diameter * of Inhibition Zone, mm
*E. coli* ATCC 25922	1	Ag ^a^	9.33 ± 0.57
	Ag + Chitosan 1%		
	2	Ag ^b^ + Chito	5.33 ± 0.57
	3	Ag ^c^ + Chito	5.66 ± 0.57
	4	Ag ^d^ + Chito	5.33 ± 0.57
*S. aureus* ATCC 25923	1	Ag ^a^	13.0 ± 1
	2	Ag ^b^ + Chito	8.33 ± 0.57
	3	Ag ^c^ + Chito	8.0
	4	Ag ^d^ + Chito	7.0

Chemical deposition of silver with the concentration in coating (^a^)—18.34%; (^b^)—9.1%; (^c^) −1.72%; (^d^) −1.03% and chitosan 1%. (*) Average ± StDev.

## Data Availability

The original contributions presented in the study are included in the article; further inquiries can be directed to the corresponding authors.
